# Pelvic Soft Tissue Aggressive Angiomyxoma

**DOI:** 10.5334/jbsr.2230

**Published:** 2020-09-18

**Authors:** Sofia Amante, Rafaela Sousa, Rui Amaral

**Affiliations:** 1 Hospital do Divino Espírito Santo de Ponta Delgada, PT

**Keywords:** Aggressive Angiomyxoma, Benign Tumors, Pelvic Neoplasms, Perineum, MRI

## Abstract

**Teaching Point:** Aggressive angiomyxoma is a rare and benign soft tissue lesion, characterized by a “whorled” or “layering” appearance on T2-weighted and post-gadolinium magnetic resonance.

## Case Report

A 49-year-old healthy woman presented to the hospital with a painless right gluteal swelling that progressively increased in size over the past two years and caused discomfort when cycling. No history of trauma was indicated.

Rectal and vaginal examinations were normal. Laboratory findings, including tumor markers, were unremarkable.

**Figure 1:**
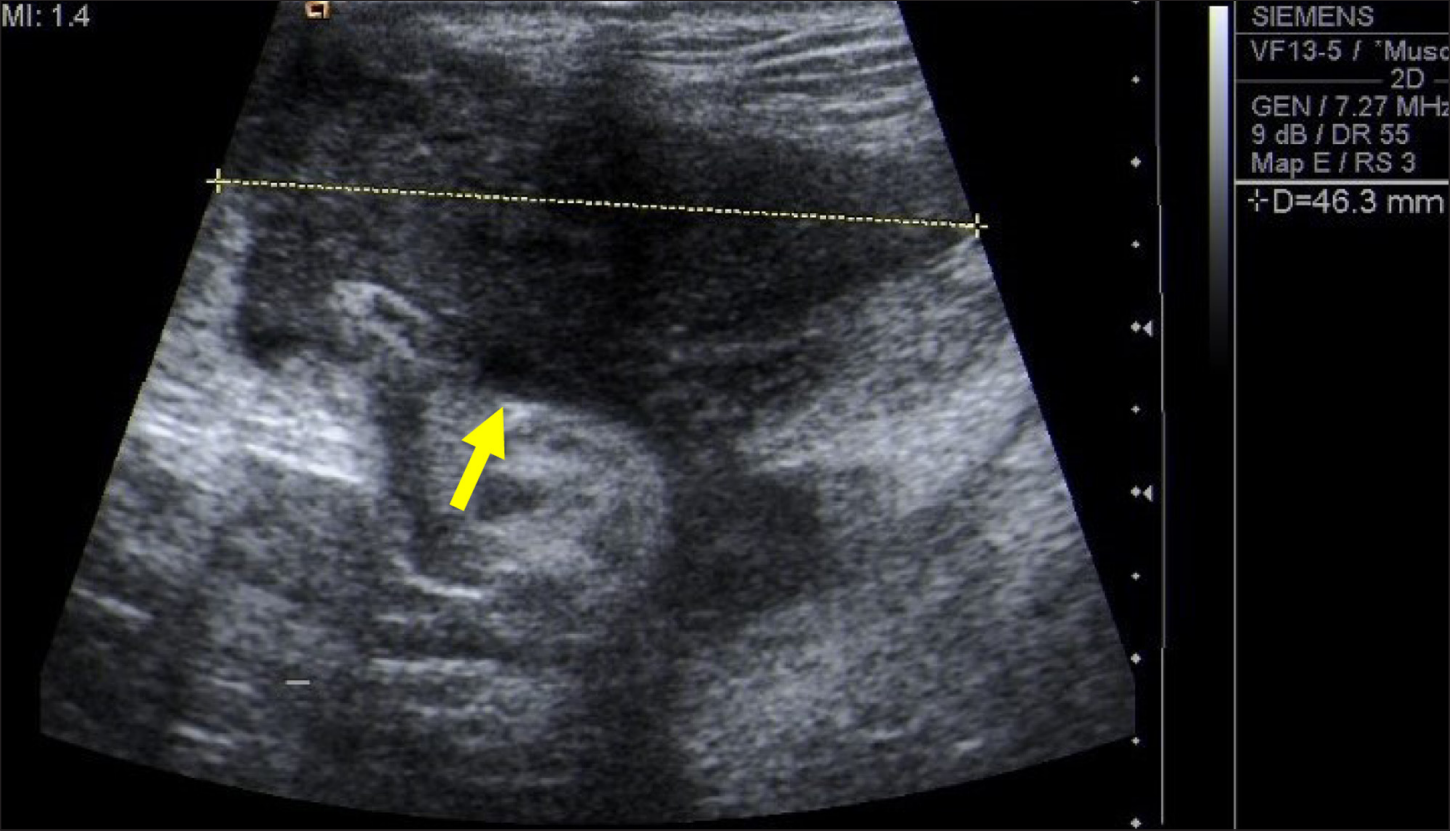


On ultrasound, a vascularized hypoechogenic and heterogeneous mass was found posterior to the ischio-pubic bone (Figure 1).

**Figure 2:**
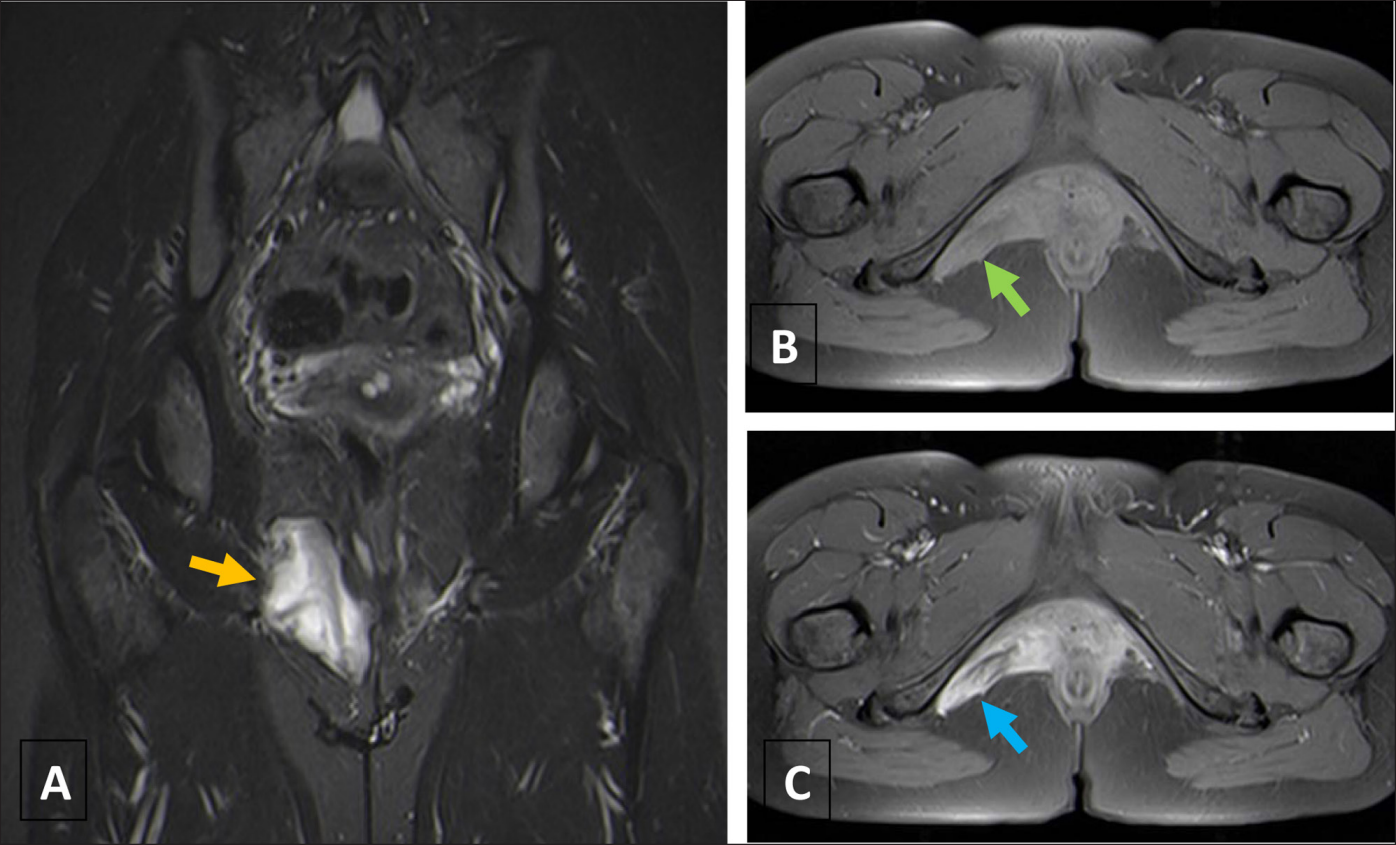


Magnetic resonance imaging (MRI) showed a mass in the right ischiorectal fossa, with well-defined limits and digitiform projections, measuring at least 6.8 × 5.8 × 3 cm. It shaped without invading adjacent structures, such as the right levator ani and the adductor muscles, the vagina, and the anal canal. The lesion showed iso-signal intensity to muscle on T1-weighted images (Figure 2B) and heterogeneous high-signal intensity on T2-weighted images, with multiple hypointense linear strands causing a layering pattern (Figure 2A). Intense enhancement and layering appearance were visualized after gadolinium (Figure 2C).

**Figure 3:**
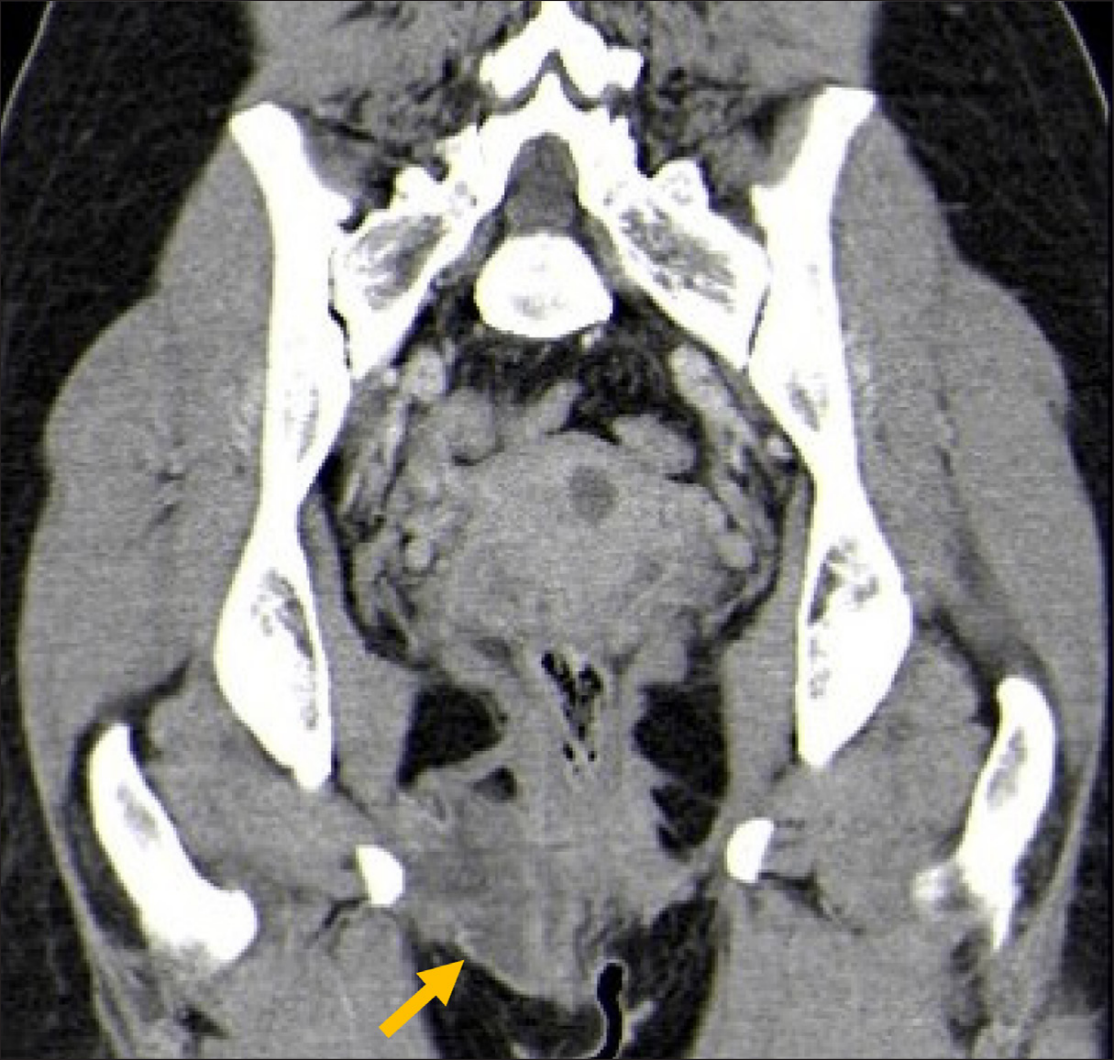


Computed tomography (CT) was consistent with MRI features, demonstrating an expansive lesion of low density with heterogeneous enhancement extending from the vulvar region to the right ischiopubic bone (Figure 3).

A core needle biopsy and subsequent surgical resection were performed, diagnosing an aggressive angiomyxoma (AA).

The patient remains asymptomatic without imaging recurrence several months later.

## Discussion

Aggressive angiomyxoma is a rare mesenchymal tumor with a predilection for the perianal and pelvic region. Characteristically, it’s a slow-growing benign tumor, with locally aggressive and infiltrative behavior. The majority of cases occur in premenopausal women, with a female-to-male ratio of 6:1. Patients frequently present with a painless and indolent growing mass, achieving large dimensions on presentation.

MRI is the best imaging technique for characterization and extent assessment, having an essential role in surgical planning. Typically, the lesion demonstrates iso to low-signal on T1-weighted images and heterogeneous high-signal on T2-weighted sequences [1]. Post-gadolinium studies reveal intense enhancement and a classic “whorled” or “layering” appearance, with alternating hyper- and hypo-intense linear areas, which is also depicted on T2-weighted sequences [1]. This pattern is caused by the presence of collagen fibrils in the myxoid tissue and is highly suggestive of AA, occurring in at least 83% of patients.

On CT, AA is hypo or isodense and has variable enhancement after contrast [1].

The differential diagnosis includes soft-tissue tumors with a myxoid pattern, such as angiomyofibroblastoma, soft-tissue sarcomas, pelvic myxoma, and a solitary fibrous tumor. These lesions don’t exhibit a whorled/layering appearance on MRI, a feature that contributes to differentiating them from AA [1].

Complete surgical resection is the treatment of choice. Unfortunately, the infiltrative nature of AA is associated with difficult complete excisions, so that about 70% of cases recur in three years.
